# VAT=TAAT-SAAT: Innovative Anthropometric Model to Predict Visceral Adipose Tissue Without Resort to CT-Scan or DXA

**DOI:** 10.1002/oby.20033

**Published:** 2012-09-19

**Authors:** Hanen Samouda, Anne Dutour, Kathia Chaumoitre, Michel Panuel, Olivier Dutour, Frédéric Dadoun

**Affiliations:** 1 Public Health Department, Health Studies Center, Center de Recherche Public-SantéL-1445 Strassen, Luxembourg; 2 Endocrinology and Nutrition Department, Hôpital NordAP-HM, F-13915 Marseille Cedex 20, France; 3 INSERM Department Université de la Méditerranée, Faculté de Médecine TimoneF-13385 Marseille, France; 4 Medical Imaging Department, Hôpital NordAP-HM, F-13915 Marseille Cedex 20, France; 5 Research Unit Department 6578 « Adaptabilité bioculturelle »—CNRS/Université de la Méditerranée. Faculté de Médecine—Secteur NordF- 13916 Marseille Cedex 20, France; 6 Department of Anthropology, University of Toronto19 Russel Street, Toronto, ON, Canada

## Abstract

**Objective:**

To investigate whether a combination of a selected but limited number of anthropometric measurements predicts visceral adipose tissue (VAT) better than other anthropometric measurements, without resort to medical imaging.

**Hypothesis:**

Abdominal anthropometric measurements are total abdominal adipose tissue indicators and global measures of VAT and SAAT (subcutaneous abdominal adipose tissue). Therefore, subtracting the anthropometric measurement the more correlated possible with SAAT while being the least correlated possible with VAT, from the most correlated abdominal anthropometric measurement with VAT while being highly correlated with TAAT, may better predict VAT.

**Design and Methods:**

BMI participants' range was from 16.3 to 52.9 kg m^−2^. Anthropometric and abdominal adipose tissues data by computed tomography (CT-Scan) were available in 253 patients (18-78 years) (CHU Nord, Marseille) and used to develop the anthropometric VAT prediction models.

**Results:**

Subtraction of proximal thigh circumference from waist circumference, adjusted to age and/or BMI, predicts better VAT (Women: VAT = 2.15 × Waist C − 3.63 × Proximal Thigh C + 1.46 × Age + 6.22 × BMI − 92.713; *R*^2^ = 0.836. Men: VAT = 6 × Waist C − 4.41 × proximal thigh C + 1.19 × Age − 213.65; *R*^2^ = 0.803) than the best single anthropometric measurement or the association of two anthropometric measurements highly correlated with VAT. Both multivariate models showed no collinearity problem. Selected models demonstrate high sensitivity (97.7% in women, 100% in men). Similar predictive abilities were observed in the validation sample (Women: *R*^2^ = 76%; Men: *R*^2^ = 70%). Bland and Altman method showed no systematic estimation error of VAT.

**Conclusion:**

Validated in a large range of age and BMI, our results suggest the usefulness of the anthropometric selected models to predict VAT in Europides (South of France).

## Introduction

Visceral adipose tissue (VAT) excess is a very important independent predictor, related to body composition, of metabolic syndrome, cardiovascular disease, and mortality independent of BMI (1-5). Computed tomography scan (CT-scan) and magnetic resonance imaging (MRI) provide a reproducible and accurate measurement of VAT. Both techniques can measure the volume of VAT in the whole peritoneal cavity, but a single slice measurement by either CT-scan or MRI is generally used as a criterion measurement of VAT (5-14). Moreover, several authors have estimated visceral fat by Dual-energy X-ray Absorptiometry (DXA) (6-8, 15-17). DXA provides actually precise measurements of total and segmental body fat and can be used to measure trunk and/or abdominal fat. However, the measurement of trunkal or abdominal fat by DXA does not distinguish between VAT and SAAT, and therefore does not predict VAT with accuracy (6-8, 15-17). Recently, specific DXA measurements centered on the visceral abdominal region have been shown to be more accurate (6-8, 13, 15, 17). Nevertheless, these techniques are often prohibitive due to cost, accessibility, and/or radiation delivery. On the other hand, simple anthropometric measurements and especially waist circumference (WC), but also in some studies sagittal abdominal diameter (SAD), are widely used to assess abdominal fat deposition, and/or all-cause morbidity or mortality, in the clinical setting or in studies involving large samples of subjects (6-10, 14, 18-20). Many investigators are even ready to do 2 measurements such as waist and hip circumferences to calculate waist-to-hip ratio (WHR), waist and thigh circumferences to determine waist-to-thigh ratio or sagittal abdominal diameter and thigh circumference to calculate Kahn index (21, 22). However, these measurements and ratios assess total abdominal adipose tissue (TAAT), cannot therefore differentiate between visceral and subcutaneous abdominal adipose tissue (SAAT) and remain less accurate than CT-scan or MRI. In other studies, some investigators have proposed to combine multiple anthropometric measurements and/or DXA parameters (6, 15, 16, 23) to predict visceral adipose tissue with more accuracy, but such low-cost methods were time consuming and not always accurate.

Our aim was to investigate and validate whether association of a limited but selected number of anthropometric measurements should better predict VAT, without resort to medical imaging.

### Hypothesis

Abdominal anthropometric measurements being total abdominal adipose tissue (TAAT) indicators and global measures of VAT and SAAT taken together, we hypothesized that subtracting the anthropometric measurement, the more correlated possible with SAAT while being the least correlated possible with VAT, from the most correlated abdominal anthropometric measurement with VAT while being highly correlated with TAAT, may differentiate VAT and SAAT and better predict VAT.

Therefore, we aimed to:

1. Identify distinctive anthropometric measurements of VAT and SAAT:
  - the abdominal anthropometric measurement the most correlated with VAT, while being highly correlated with TAAT;
  - the anthropometric measurement the more correlated possible with SAAT, while being the least correlated possible with VAT;
2. Combine these measurements, according to the hypothesis, to develop the most accurate anthropometric VAT prediction in the form of the model VAT = TAAT-SAAT;
3. Validate the concept VAT = TAAT-SAAT by DXA;
4. Validate our anthropometric developed models in a 2nd patient's sample.

## Methods and Procedure

For medical reasons, body composition of 253 patients (148 women, 105 men) from the Department of Endocrinology, Metabolism and Nutrition (CHU Nord, Marseille, France) has been assessed. Participants were metabolically settled type 1 diabetics or obese subjects but were free of diseases liable to affect body composition or influence anthropometric measures (type 2 diabetes, Prader Willi syndrome, leptin deficiency, Laurence Moon Biedl syndrome, AIDS, glucocorticoid treatment, unsettled thyroid disease, abdomen tumor). Total and subcutaneous abdominal adipose tissues and visceral adipose tissue were available by CT-Scan for all of the patients: Sample 1 (*n* = 114, first year of the study, development of VAT anthropometric models in relation to CT-scan) and Sample 2 (*n* = 139, second year of the study, validation of VAT anthropometric models in relation to CT-scan). DXA performed in sample 1 were useful to validate the “VAT = TAAT-SAAT” anthropometric concept. Abdominal anthropometric circumferences and diameters, limb circumferences and skinfolds thickness were evaluated within the framework of our study for each participant. BMI and age ranges were from 16.32 to 52.94 kg m^−2^, respectively from 18 to 78 years. The study was approved by the local committee for ethics. Informed consent was obtained from each participant.

### Criterion measurement of VAT, SAAT and TAAT by CT-scan

A single 10-mm axial slice was acquired by CT-scan at the L4-L5 lumbar vertebrae level using a General Electric Medical System High Speed CTI® tomodensitometer (40 Kv, 220 mA, acquisition time: 2 s), as previously described by Smith et al. (24). Patients were in supine position, their arms above the head. VAT, SAAT and TAAT surfaces (cm^2^) were calculated with the image analysis software linked to the CT-scan equipment. To distinguish adipose tissue from muscle and bone tissues, the fixed CT-Scan attenuation range from −190 to −30 Hounsfield Units for adipose tissue was applied, as defined by Sjöstrom etal. (11) and Kvist etal. (12).

All adipose tissue pixels inside the line tracing the skin abdominal circumference were regarded as TAAT, as showed first (24). All adipose tissue pixels inside the line starting from the linea alba, bisecting the rectus abdominus, the internal oblique, the iliacus and surrounding the peritoneum, were defined as VAT. SAAT was defined by all adipose tissue pixels outside this latter line (24).

### Anthropometry

In addition to weight and stature, 24 anthropometric measurements were performed in all patients by a single investigator (H.S.) according to Lohmann (25), within the framework of our study, to provide anthropometric estimation of:

#### Abdominal adiposity

Waist circumference (Waist C) was measured midway between the lower rib and the iliac crest on the midaxillary line; and at the umbilicus. The measurement of abdominal diameters was made at the same level (the approximate location was between the 4th and the 5th lumbar vertebrae). The sagittal abdominal diameter (SAD) was measured with two different types of portable calliper: straight and curved blade callipers. The transversal abdominal diameter (TAD) was measured with a straight blade calliper. All abdominal measurements were performed with the subjects standing and supine, except the curved blade calliper SAD (standing). Measurements were made at various levels to test which way of measurement is more associated with VAT. Abdominal, pectoral and subscapular skinfolds thicknesses were assessed with the thickness caliper.

#### Limb adiposity (on the subject standing)

Thigh circumferences were measured at three levels: proximal [(proximal thigh C); a distal positioning of the measuring tape to the gluteal crease and around the thigh], [distal (distal thigh C); a proximal positioning of the measuring tape to the femoral epicondyle] and [mid-thigh (mid thigh C); an horizontally positioning of the measuring tape halfway between inguinal ligament and the proximal patella side]. Hip [(Hip C); the greater perimeter at the major trochanter level, on the pectineal line of pubis], calf [(Calf C); an horizontally positioning of the measuring tape halfway between knee (patella) and ankle (lateral malleolus), knee [(knee C); at the patella level], ankle [(ankle C); perimeter linking lateral and medial malleolus], arm [(Arm C); an horizontally positioning of the measuring tape halfway between acromion and olecranon], forearm [(Forearm C); an horizontally positioning of the measuring tape halfway between olecranon and pisiform], elbow [(elbow C); perimeter around elbow joint] and wrist [(wrist C); perimeter around wrist joint] circumferences were also performed. Tricipital, bicipital, supra-iliac, and thigh skinfolds thickness were assessed with the thickness caliper.

#### Ratios

Body mass index (BMI) and waist-to-hip ratio (WHR) were calculated.

### Total body fat and regional/segmental fat by DXA

Total body fat and regional body fat measurements were measured by DXA, with the Hologic®QDR4500W densitometer:

- Six conventional predefined fat areas: head, trunk (trunk fat), arms (left arm fat and right arm fat) and legs (left leg fat and right leg fat);
- Six specific fat areas manually defined from skeletal landmarks and using standard cut lines, with the purpose to correlate either with visceral fat or with subcutaneous fat: 1. [abdominal + pelvic fat] area delineated by a horizontal line linking the last thoracic rib lower borders through the rachis; two vertical lines linking the shoulders (at the glenoid fossa) with the thighs and two angled lines bisecting the femoral necks below the pelvis. 2. [Thigh Fat] area delineated by the angled line bisecting the femoral neck below the pelvis, a vertical line lateral to the thigh and going on until the knee, a horizontal line going through the knee and a vertical line linking the knee with the pelvis. 3. [Central Fat] area delineated by the horizontal line linking the last thoracic rib lower borders through the rachis; a horizontal line above the pelvis (just above the crest of the ilium) and two vertical lines linking these boundaries at the level of the external edge of the rib cage. 4. [Intra-Pelvic Fat] area delineated by the triangle linking the posterior superior iliac spines with the pubis symphis. 5. The [central + intra-pelvic fat] area was defined as the sum of the [central fat] and the [intra-pelvic fat] areas. 6. The [peripheral abdominal fat] area was obtained by subtracting the [central + intra-pelvic fat] area from the [abdominal + pelvic fat] area.

### Design of VAT anthropometric prediction models and statistical analysis

Statistical analysis was performed using SPSS® for Windows Version 17.0. All descriptive data were expressed as mean ± sd. Data of the two volunteers groups were analyzed. The first set of data (sample 1, *n* = 114, first year of the study) was analyzed to identify distinctive anthropometric measurements of VAT and SAAT and to develop anthropometric VAT models against CT-scan (gold standard). Pearson correlation coefficients (*R*) were established between anthropometry and CT-Scan to identify distinctive anthropometric measurements of VAT and SAAT (in particular anthropometric measurements the more correlated possible with VAT while being highly correlated with TAAT, as well as the ones that are the more correlated possible with SAAT while being the least correlated possible with VAT). Multiple linear regressions were developed with VAT as a dependent variable and a maximum of two anthropometric parameters as independent variables, adjusted to age, gender, weight and/or BMI. According to our hypothesis “VAT = TAAT-SAAT,” we associated, as independent variables to predict VAT, the anthropometric measurement the more correlated possible with SAAT while being the least correlated possible with VAT, with the abdominal anthropometric measurement the more correlated possible with VAT and highly associated to TAAT. *R*, *R*^2^, and SEE (standard error estimation, cm^2^) were calculated. To be included in models, each variable required firstly not be collinear with another and secondly to significantly contribute (significant *r*_partial_). We also performed a no controlled stepwise regressions to verify this empirical parameters selection: a forward selection, starting with no variables in the model, trying out the variables one by one and including them if they are statistically significant.

Moreover, we compared these models with: 1. the anthropometric measurement the more correlated possible with VAT, adjusted to age and BMI; 2. others models combining two anthropometric measurements both highly correlated with VAT (±age ±BMI). We tested the possible pair of combinations of the three single more correlated anthropometric variables with VAAT. We evaluated variances explained par models and collinearity in each of them: 1. serious collinearity problems if condition index > 30; 2. possible collinearity if condition index > 15; 3.collinearity if two or more variables with a variance proportion ≥0.5, variance inflation factor (VIF) ≥4.0 and tolerance <0.25 (26).

We assessed, on the other hand, the accuracy of VAT prediction for selected models by using the Bland and Altman plots (27). To test the relevance of the selected models for the diagnosis of VAT excess in a clinical setting, we determined the sensitivity (Se), specificity (Sp), positive and negative predictive value (PPV, NPV) of the models for predicting VAT > 130 cm^2^ (28).

The same procedure was applied with conventional and specific DXA fat areas to validate the anthropometric concept. Finally, we tested, in the sample 1, the equations published in the literature (*R*^2^ assessment).

Intra- and inter observer (s) precision of anthropometric measurements in the selected models were assessed [intraclass correlation coefficient (ICC); coefficient of variation (CV); Bland and Altman method]. Apart from the principal investigator, three others observers took part in the inter observers variability assessment, amongst 30 participants.”

The second set of data (sample 2, *n* = 139, second year of the study) was a sample to test our anthropometric models, against CT-scan [*R*, *R*^2^, and SEE (cm^2^)].

## Results

### Subject characteristics

The characteristics of the 253 volunteers (sample 1, VAT assessment sample: *N* = 71 women and 43 men; sample 2, VAT validation sample: *N* = 77 women and 62 men) are presented in Table 1.

**TABLE 1:** Subject characteristics: age, anthropometry, DXA and CT-scan

	Sample 1: Assessment model of VAT		Sample 2: Validation of VAT model	
		
	71 Women	43 Men	77 Women	62 Men
	Mean ± sd; Min-Max	Mean ± sd; Min-Max	Mean ± sd; Min-Max	Mean ± sd; Min-Max
**Age (years)**	46.55 ± 15.42; 20-77	46.95 ± 15.63; 18-78	42.78 ± 13.47; 18-69	43.74 ± 12.55; 18-68
**Weight (kg)**	80.31 ± 19.17; 43.9-126.2	91.29 ± 19.97; 61.1-144.5	83.81 ± 18.65; 50.2-128.2	102.85 ± 25.09; 56.8-153
**BMI (kg m^−2^)**	31.37 ± 7.21; 16.32-50.38	30.14 ± 6.76; 18.86-47.80	32.16 ± 6.43; 19.25-47.96	33.72 ± 8.19; 18.55-52.94
**Waist C (cm)**	94.64 ± 17.42; 59-134	101.05 ± 16.87; 69-134	96.26 ± 15.43; 65-136	108.49 ± 19.21; 68-152
**SAD (cm)**	21.66 ± 6.86; 9-38	24.7 ± 6.85; 11.5-37.8	24.51 ± 6.52; 10.2-43	28.36 ± 7.01; 13-41.8
**Proximal thigh C (cm)**	62.06 ± 7.9; 39-80	57.31 ± 7.47; 44-78	65.75 ± 7.13; 46.5-89	62.56 ± 8.95; 43-81
**Total body fat_DXA_ (kg)**	32.30 ± 13.36; 3.67-65.79	23.20 ± 12.23; 3.19-51.44	-	-
**Trunk fat_DXA_ (kg)**	15.96 ± 8.55; 1.54-38.8	13.6 ± 8.11; 1.5-32.63	-	-
**Central fat_DXA_ (kg)**	4.26 ± 2.47; 0.28-11.12	4.33 ± 2.5; 0.3-9.93	-	-
**Intra-pelvic fat_DXA_ (kg)**	1.45 ± 0.76; 0.24-4.74	1.17 ± 0.59; 0.1-2.43	-	-
**Left thigh fat _DXA_ (kg)**	4.37 ± 1.61; 0.38-10.13	2.27 ± 1.25; 0.15-6.03	-	-
**VAT_CT-scan_ (cm^2^)**	148.88 ± 88.44; 13.45-377.35	196.40 ± 103.71; 33.74-442.48	142.11 ± 80.15; 18.03-452.18	215.88 ± 111.16; 34.29-585.87
**SAAT_CT-scan_ (cm^2^)**	402.22 ± 175.54; 49.17-774.75	285.64 ± 163.42; 40.73-662.93	441.57 ± 158.49; 126.75-776.32	360.26 ± 192.75; 32.95-817.36

Abbreviations: BMI, body mass index; Waist C, waist circumference; SAD, sagittal abdominal diameter (straight blade calliper, subject supine); Proximal Thigh C, proximal thigh circumference; VAT, visceral adipose tissue; SAAT, subcutaneous abdominal adipose tissue.

### Single parameter estimation of visceral adipose tissue (VAT), subcutaneous abdominal adipose tissue (SAAT) and total abdominal adipose tissue (TAAT)

Standing sagittal abdominal diameter (SAD), measured with a curved blade caliper, appears to be the best single anthropometric measurement for the assessment of VAT in both genders, Waist C (measured midway between the lower rib and the iliac crest on the midaxillary line) being nearly as powerful. Both SAD and Waist C are highly correlated with TAAT but higher correlation is observed with Waist C.

Proximal thigh circumference (Proximal Thigh C) is the anthropometric measurement the more correlated possible with SAAT, while being the least correlated possible with VAT

Proximal thigh C is also clearly less correlated with TAAT than waist.

Proximal thigh C is rather correlated with SAAT (*R*: Women = 0.72; Men = 0.74), whereas Waist C is rather correlated with TAAT (Table 2).

**TABLE 2:** Pearson's correlation coefficients of TAAT, VAT, and SAAT to anthropometric measurements

Measurement	*R*	*R*	Measurement	*R*	Measurement
	VAT	TAAT		SAAT	TAAT
A. WOMEN					
SAD	0.847	0.848	Hip C	0.937	0.909
Waist C	0.835	0.952	Waist C	0.897	0.952
WHR	0.814	0.705	TAD	0.876	0.900
TAD	0.736	0.900	SAD	0.828	0.848
Abdominal skinfold	0.691	0.812	Arm C	0.813	0.783
Hip C	0.639	0.909	Supra-iliac skinfold	0.784	0.773
Pectoral skinfold	0.603	0.710	Abdominal skinfold	0.776	0.812
Subscapular skinfold	0.602	0.758	Subscapular skinfold	0.749	0.758
Supra-iliac skinfold	0.558	0.773	Bicipital skinfold	0.740	0.754
Arm C	0.541	0.783	Proximal thigh C	0.718	0.597
Bicipital skinfold	0.508	0.754	Tricipital skinfold	0.706	0.680
Tricipital skinfold	0.471	0.680	Pectoral skinfold	0.682	0.710
Forearm C	0.456	0.523	Mid thigh C	0.575	0.456
Calf C	0.239	0.376***	WHR	0.565	0.705
Proximal thigh C	0.211	0.597	Distal thigh C	0.543	0.471
Distal thigh C	0.210	0.471	Forearm C	0.495	0.523
Mid thigh C	0.108	0.456	Calf C	0.399	0.376***
Thigh skinfold	NS	0.340**	Thigh skinfold	0.376*	0.340**
B. MEN					
SAD	0.833	0.774	Waist C	0.882	0.958
Waist C	0.812	0.958	Hip C	0.867	0.823
TAD	0.810	0.918	TAD	0.825	0.918
WHR	0.778	0.700	Arm C	0.802	0.822
Arm C	0.625	0.822	Supra-iliac skinfold	0.777	0.792
Bicipital skinfold	0.612	0.721	Pectoral skinfold	0.774	0.743
Subscapular skinfold	0.557	0.698	SAD	0.747	0.774
Pectoral skinfold	0.583	0.743	Subscapular skinfold	0.728	0.698
Supra-iliac skinfold	0.523	0.792	Abdominal skinfold	0.727	0.794
Hip C	0.519	0.823	Proximal thigh C	0.736	0.610
Tricipital skinfold	0.513	0.623	Calf C	0.716	0.633
Distal thigh C	0.453	0.684	Tricipital skinfold	0.695	0.623
Abdominal skinfold	0.435**	0.794	Bicipital skinfold	0.651	0.721
Forearm C	0.443	0.650	Distal thigh C	0.710	0.684
Calf C	0.330	0.633	Forearm C	0.667	0.650
Proximal thigh C	0.238	0.610	Mid thigh C	0.634	0.530
Mid thigh C	0.216	0.530	WHR	0.532	0.700

Abbreviations: SAD, sagittal abdominal diameter (straight blade calliper, subject supine); Waist C, waist circumference; WHR, waist to hip ratio; TAD, transversal abdominal diameter (straight blade calliper, subject supine); Hip C, hip circumference; Arm C, arm circumference; Forearm C, forearm circumference; Calf C, calf circumference; Proximal Thigh C, proximal thigh circumference; Distal Thigh C, distal thigh circumference; Mid Thigh C, mid thigh circumference; VAT, visceral adipose tissue; SAAT, subcutaneous abdominal adipose tissue. All *P* values < 0.05 except *0.01; **0.004, ***0.001.

### Assessment of visceral adipose tissue using models that combine multiple parameters

Multiple linear regressions showed that the two anthropometric measurements (SAD or Waist C/Proximal Thigh C as independent variables) combined with age and/or BMI provide a good prediction of VAT (Table 3). Model including SAD explains ∼86% of VAT variance in women, ∼79% in men (Annex C). Model combining Waist C_,_ Proximal Thigh C, Age and BMI explains ∼84% of VAT variance in women, ∼80.5% in men. We made the choice to substitute SAD for Waist C because of the easier feasibility of the waist circumference measurement. Therefore, our selected anthropometric models are:

1. In women: VAT = 2.15 × Waist C − 3.63 × proximal thigh C + 1.46 × age + 6.22 × BMI − 92.713 [Model: *R*^2^ = 0.836; SEE = 36.88; *P* < 10^−4^. Waist C: *r*_partial_ = 0.313; *P* = 0.009. Proximal thigh C: *r*_partial_ = −0.463; *P* < 10^−4^. Age: *r*_partial_ = 0.433; *P* < 10^−4^. BMI: *r*_partial_ = 0.355; *P* = 0.003]
2. In men: VAT = 6 × Waist C − 4.41 × proximal thigh C + 1.19 × Age − 213.65 [Model: *R*^2^ = 0.803; SEE = 47.73; *P* < 10^−4^. Waist C: *r*_partial_ = 0.819; *P* < 10^−4^. Proximal thigh C: *r*_partial_ = −0.408; *P* = 0.048. Age: *r*_partial_ = 0.305; *P* = 0.008]. BMI's *r*_partial_ is no significant in men, but adding BMI improves *R*^2^ in the model (0.817) (Annex B). Both selected models showed no collinearity problem [(condition index < 15, variance proportion < 0.5, VIF < 4, tolerance > 0.25) (Annex A, B)].

**TABLE 3:** Assessment and validation of VAT using models that combine multiple parameters

	*R*	*R*^2^	SEE cm^2^	Parameters included
A. WOMEN				
*VAT Estimation*				
Anthropometry	0.927	0.859	34.25	SAD / Proximal Thigh C / Age / BMI
	0.914	0.836	36.88	Waist C / Proximal Thigh C / Age / BMI
DXA_conventional_	0.890	0.792	41.55	Trunk Fat / Left Leg Fat / Age / BMI
DXA_specific_	0.906	0.821	38.50	Central + Intra-Pelvic Fat / Left Thigh Fat / Age / BMI
Anthropometry + DXA_conventional_	0.928	0.862	34.38	Trunk fat / Left Arm Fat + Right Arm Fat / Proximal Thigh C / SAD / Age / BMI
Anthropometry + DXA_specific_	0.936	0.876	32.51	Central + Intra-Pelvic Fat / Left Thigh Fat / SAD / Proximal Thigh C / Age / BMI
*VAT Validation* – Anthropometry	0.872	0.760	31.94	Waist C / Proximal Thigh C / Age / BMI
B. MEN				
VAT estimation				
Anthropometry	0.890	0.792	49.78	SAD /proximal thigh C/Age/BMI
	0.896	0.803	47.73	Waist C/proximal thigh C/age
DXA_Conventional_	0.877	0.768	52.48	Trunk fat/right leg fat/age/BMI
DXA_Specific_	0.881	0.776	51.65	Central fat/right thigh fat/age/BMI
Anthropometry + DXA_Conventional_	0.916	0.840	44.88	Trunk fat/left arm fat + right arm fat + left leg fat + right leg fat/proximal thigh C/SAD /age/weight
Anthropometry + DXA_Specific_	0.932	0.869	40.51	Central fat/right thigh fat/proximal thigh C/SAD/age/weight
*VAT Validation* − anthropometry	0.838	0.702	50.64	Waist C/proximal thigh C/age

Abbreviations: VAT, visceral adipose tissue; DXA, dual-energy X-ray absorptiometry; SAD, sagittal abdominal diameter; Proximal Thigh C, proximal thigh circumference; BMI, body mass index; Waist C, waist circumference. All *P* values < 0.05.

For the selected models, intra observer's ICC was equal to 1. Inter observer's ICC values were, respectively, 0.93, 0.98 and 0.99 for Proximal Thigh circumference; 0.99, 0.99, and 1 for Waist circumference. For proximal thigh circumference, CV was equal to 0.01 for intra observer measurement and equal to 0.022 for interobserver measurement.

Moreover, the Bland and Altman analysis showed no systematic estimation error between observers.

Comparisons between our newly developed anthropometric models and: 1. The anthropometric measurement the more correlated possible with VAT (adjusted to age and BMI), as well as 2. The associations of the two anthropometric measurements the more correlated with VAT (±age ±BMI), showed less favorable results, in explained variance, errors and collinearity, than the anthropometric models which were developed (Annex A, B):

1. “Waist C + age ± BMI model”: In women: VAT = 2.439 × Waist C + 2.089 × Age + 2.510 × BMI − 257.946. (*R*^2^ = 0.791; SEE = 41.30; *P* < 10^−4^). In men: VAT = 4.59 × Waist C + 2.188 × Age − 370.379. (*R*^2^ = 0.764; SEE = 51.63; *P* < 10^−4^). BMI has been removed from models when collinear and no significant
2. Combination of the two anthropometric measurements the more correlated with VAT: 2.1. Variances explained par models, in women: [Waist C, SAD, age ± BMI (*R*^2^ = 0.807/0.811)]; [SAD, WHR, age ± BMI (*R*^2^ = 0.804/0.834)]; [Waist C, WHR, age ± BMI (*R*^2^ = 0.796/0.817)]. In men: [Waist C, SAD, age ± BMI (*R*^2^ = 0.767/0.768)]; [SAD, TAD, age ± BMI (*R*^2^ = 0.311/0.749)]; [Waist C, TAD, age ± BMI (*R*^2^ = 0.764/0.765)]. 2.2. Each model had a condition index > 15, a variance proportion > 0.5, a VIF > 4, tolerance < 0.25, therefore a collinearity problem.

On the other hand, successive selected parameters by the stepwise regression (a forward selection) were:

- in women: SAD (*R*^2^ = 0.718), age (age and SAD: *R*^2^ = 0.799), BMI (age, SAD and BMI: *R*^2^ = 0.816), proximal thigh C (age, SAD, BMI, and proximal thigh C: *R*^2^ = 0.859), TAD (age, SAD, BMI, proximal thigh C, and TAD: *R*^2^ = 0.876) and knee circumference (age, SAD, BMI, proximal thigh C, TAD, and knee circumference: *R*^2^ = 0.884);
- in men: Waist C (*R*^2^ = 0.695), age (age and Waist C: *R*^2^ = 0.76), proximal thigh C (age, Waist C and proximal thigh C: *R*^2^ = 0.80) and, finally, distal thigh C (age, waist C, proximal thigh C and distal thigh C: *R*^2^ = 0.83).

Combining conventional and/or specific abdominal respectively leg fat areas by DXA confirms our findings, but appears less accurate than our selected anthropometric models (Table 3). Combination of two specific DXA measurements with anthropometry improves VAT assessment moderately (∼87% of VAT variance), but requires the measurement of many parameters (Table 3).

### Ability of selected anthropometric models to assess VAT excess (VAT > 130 cm^2^)

The selected models demonstrate high sensitivity (97.7% in women, 100% in men) and predictive values (PPV%: 91.3 in women, 90.9 in men; NPV%: 96 in women, 100 in men) in both gender, but theirs specificities are not optimal, especially in men (75% in men, 85.7% in women).

### Comparison of the selected models/equations to previously published models/equations

None of previously published models, tested in our study population, did provide a better prediction of VAT than our anthropometric models described above.

Anthropometric equations proposed by Seidell et al. (19) and by Desprès et al.(18) retain in our population a close predictive power to the correlation obtained in the original studies. Similar findings were observed in our women's group with the results described by Treuth et al. (15) who have combined anthropometry and DXA in women (Table 4).

**TABLE 4:** Comparison of the selected models/equations to previously published models/equations

Reference	Parameters included		*R*^2^ in original study	*R*^2^ in our population

	Anthropometry	DXA		
A. WOMEN				
Seidell et al. 1987 (20)	WHR/BMI/ΣSF/age	0	0.819	0.794
Svendsen et al. 1993 (17)	WHR/Log ΣSF	Abdominal fat	0.91	0.601
Treuth et al. 1995 (16)	SAD/Waist C/age	Trunk fat	0.81	0.773
B. MEN				
Seidell et al. 1987 (20)	WHR/BMI/ΣSF/age	0	0.819	0.425
Despres et al. 1991 (19)	WHR/SAD/age	0	0.766	0.747
Despres et al. 1991 (19)	SAD/Waist C/age	0	0.74	0.687

Abbreviations: DXA, dual-energy x-ray absorptiometry; WHR, waist to hip ratio; BMI, body mass index; SSF, sum of skinfolds thicknesses; SAD, sagittal abdominal diameter; Waist C, waist circumference. All *P* values < 0.05.

### Validation of our models in the 2nd sample

In the validation sample, the abilities of our anthropometric models were similar to those observed in the sample 1. The model stemming from the combination of Waist C, proximal thigh C, age, and BMI in women explains 76% of VAT variance (SEE: 31.94 cm^2^) in the 2nd women's sample. The model associating Waist C, proximal thigh C and age in men explain 70% of VAT variance (SEE: 50.64 cm^2^) (Table 3).

### Accuracy of our selected anthropometric models to predict VAT

**FIGURE 1:**
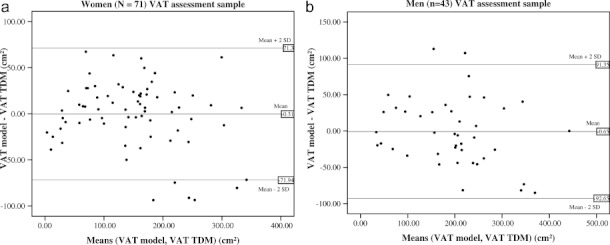
Bland and Altman plots of the differences between predicted (by our anthropometric model) and assessed (by TDM: Tomodensitometry) VAT values against means of VAT in the visceral adipose tissue assessment sample; amongst (a) 71 women and (b) 43 men.

**FIGURE 2:**
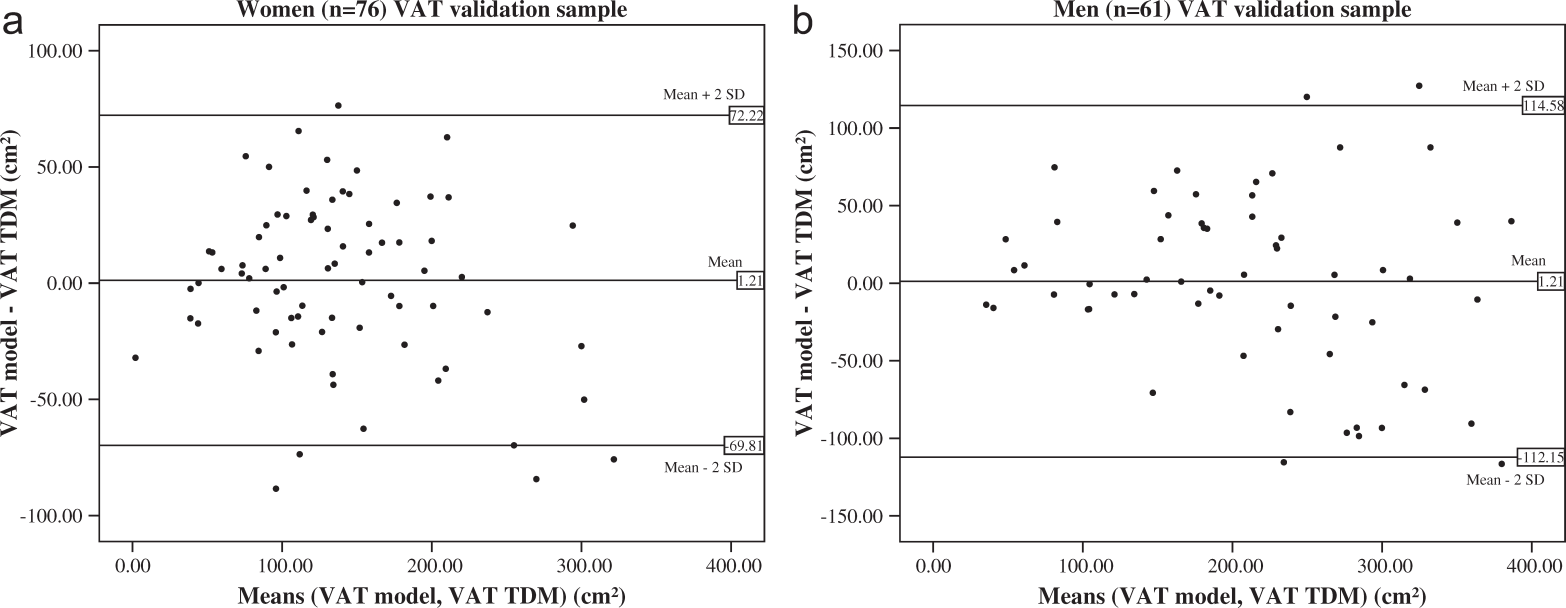
Bland and Altman plots of the differences between predicted (by our anthropometric model) and assessed (by TDM: Tomodensitometry) VAT values against means of VAT in the visceral adipose tissue validation sample; amongst (a) 76 women and (b) 61 men.

Bland and Altman method showed the absence of systematic estimation error of VAT in our assessment and validation samples (no significant correlation showed). The Bland/Altman limits of agreement (Mean ± 2 SD in cm^2^) were 1. In women: [−71.94; +71.3] in the VAT assessment sample (Figure 1a) and [−69.81; +72.22] in the VAT validation sample (Figure 2a); 2. In men: [−92.65; +91.35] in the VAT assessment sample (Figure 1b) and [−112.15; +114.58] in the VAT validation sample (Figure 2b).

## Discussion

Our study demonstrated that it is possible to easily and reliably predict VAT with only two simple and widely used anthropometric measurements (“1 abdominal − 1 leg” measurements) associated with age and BMI and without resorting to CT-scan, MRI or DXA. Despite the fact that age and BMI considerably influence body composition, and are usually collected in most epidemiological studies or in clinics, they are not frequently used in anthropometric models designed for the assessment of body composition. This concept implied the subtraction of the most correlated anthropometric measurement with SAAT, while being the least correlated possible with VAT (Thigh C), from the abdominal anthropometric measurement the most correlated to VAT while being highly correlated with TAAT (Waist C).

The modeling of these data in our study population explains 83.6 % of the variance of VAT in women (VAT = 2.15 × Waist C − 3.63 × proximal thigh C + 1.46 × Age + 6.22 × BMI − 92.713) (*R* = 0.914; *R*^2^ = 0.836; SEE = 36.88; *P* < 10^−4^); respectively 80.3% in men (VAT = 6 × Waist C − 4.41 × proximal thigh C + 1.19 × Age − 213.65) (*R* = 0.896; *R*^2^ = 0.803; SEE = 47.73; *P* < 10^−4^). We didn't include BMI in men because of its no significant contribution (*P* > 0.05), even if *R*^2^_Model_ increased (*R*^2^ = 0.817). Indeed, our VAT anthropometric prediction models design was based on the fact that to be included in the models, variables have to be significant and no collinear.

Variables selected by the no controlled stepwise regressions validated our empirical parameters selection.

We made however the choice to not include: TAD because of its collinearity with waist C; knee circumference because it is not an adipose tissue measurement and distal thigh C because of its collinearity with proximal thigh C. We replaced SAD with Waist C, straightforward to measure with a simple tool. Proximal thigh C was moreover affected by a minus sign and inversely correlated with VAT, confirming our hypothesis “VAT=TAAT-SAAT.”

On the other hand, in relation to the “Waist C + age ± BMI” model, thigh circumference makes a real significant additional contribution. Indeed, even though this improvement of R^2^ may appear modest (4.5% and 3.9%), *R*^2^, SEE, and Bland/Altman representations significant improvement, together with the absence of collinearity, the significant partial correlation, and hence the validity of our hypothesis, justify in our opinion, the little complexity added by performing an additional measurement in clinical or research settings. Moreover, in the clinical setting, many clinicians involved in weight management and metabolic care routinely perform the measurement of two anthropometric measurements (i.e. waist circumference and hip circumference), so that measuring both waist circumference and thigh circumference should not be off-putting so much. Furthermore, the measurement of thigh circumference is quite easy to perform. Associating two anthropometric abdominal measurements seems to be less optimal. Our anthropometric models showed a high level of accuracy to predict VAT, in the assessment and validation samples. VAT is a very important and independent predictor, related to body composition, of cardiovascular diseases (1-5, 29). Moreover, we obtained a good ability to assess the excess of VAT > 130 cm^2^ (28).

We agree with Kuk et al. (2007) (30) who observed that after adjustment for Waist C, Thigh C (respectively Hip C) was negatively associated with VAT. According to the authors, the combination of BMI, Thigh C (or Hip C) with Waist C is important to inform the practitioner with respect to obesity phenotypes (phenotype 1 characterized by larger Hip C or Thigh C, excess of lower-body and abdominal SAAT and consequently, lower VAT values; inversely to phenotype 2). Moreover, our results are in agreement with those of Goel et al. (2008) (31) who suggested an intra-abdominal adipose tissue (IAAT) assessment model combining Waist C, Hip C, age, sex, and BMI in Asian Indians. However, the anthropometric model used by the authors explained only 52% of the variability of IAAT and none physiological hypothesis were voiced. Furthermore, ethnic differences exist in body composition between Asians and Europides (32). Our study population is also characterized by a large age and BMI ranges, in contrast to the study of Goel et al. (2008) (31) (Age: 18-50 years; BMI: 15.3-36.9 kg m^−2^); and the size of our validation sample is more substantial. Moreover, to assess the accuracy of VAT models, we have used the Bland and Altman method (Figures 1 and 2), usually considered as an essential validation procedure even though it has rarely been applied (27). Although ideally volumetric VAT should be used as a reference method for VAT measurement, we used the L4-L5 single CT-scan slice to minimize radiation for participant.

However, our anthropometric model might not be optimal and could be refined by another level of CT-Scan slice, particularly between the third and fourth lumbar vertebra in men (33), approximately at the L3 vertebra (34) or 10 and 15 cm above L(4)-L(5) in men (35, 36), respectively 5 cm above or below L(4)-L(5) or total VAT volume in women (35, 36).Nevertheless, our study was conceived before these publications. On the other hand, all the single slices were strongly associated with total VAT and cardiovascular risk in previous studies (37).

Finally, in our study, even DXA, which is the most effective tool for measuring regional fat mass (6, 13, 15), does not generate a more precise assessment of VAT than our anthropometric models. Indeed, none of the DXA models that we have tested (several manual trunk segmentations or DXA models in accordance with our concept « VAT=TAAT-SAAT », adjusted to age and BMI) have guaranteed a better assessment of VAT than our anthropometric models, in spite of their inherent complexity and the time necessary to manually analyze multiple regional fat measurements by DXA. Furthermore, DXA analysis is too costly and not always totally accessible. Only very highly complex models combining simultaneously regional fat mass by DXA, anthropometric measurements, age and BMI showed a slightly higher performance in our study (Table 3). These models were frequently proposed in the literature to predict VAT, but never adopted in large studies because of their complexity, cost and irradiation. In this way, Svendsen et al. (16) predicted intra-abdominal adipose tissue by abdominal fat mass by DXA (region of interest comprising between L1 and L4 levels and defined by manually adjusting the lines of the right rib box), combined with WHR and the sum of three skinfolds thicknesses (*R*^2^: 0.91), in a small postmenopausal women sample. Treuth etal. (15) developed equations that combine trunk fat by DXA, sagittal diameter, waist circumference and age (*R*^2^ = 0.81) in a similar sample. The authors then divided the trunk into pelvic fat, upper and lower trunk but no specific section improved the precision of VAT estimation in women (15). Kamel et al. (17) measured three rectangular areas of abdominal tissue using DXA at the level of L2-L3 in a small group of nonobese subjects (with vertical sides of the rectangle extending to the lateral margins of the image; with vertical sides as the continuation of the lateral sides of the rib cage; and with fixed width of 15 cm; *R*: 0.83-0.9). In another small group of obese men and women, Kamel et al. (15) measured central abdominal fat by DXA (a rectangle drawn between L2 and L4, with vertical boundary on the lateral sides of the rib cage). To predict VAT, Bertin *et al.* (7) combined sagittal diameter and height by anthropometry, subcutaneous fat width and transverse internal diameter at the umbilical level by DXA (*R*^2^: 0.87 in women; 0.77 in men). In an elderly group, Snijder etal. (6) suggested using the trunk fat mass as measured by DXA to predict VAT. Finally, Hill etal. (23) combined the abdominal skinfolds and the region of interest from DXA identified by drawing a quadrilateral of 10 cm in height with the base of the box touching the top of the iliac crest and the lateral borders extending to the edge of the abdominal soft tissue. However, none of these models is easy to use.

Furthermore, our models are interesting with regard to physiology. Indeed, an opposite relationship between waist C and cardiovascular morbi-mortality on the one hand, Hip C (or thigh C) and cardiovascular morbi-mortality on the other hand, was described and explained by the health protective effect assigned to a larger Thigh C or Hip C for a given Waist C (38). This health protective effect may result either from a more important accumulation of skeletal muscle and subcutaneous fat mass in the lower body (39); or from a simultaneous increase of abdominal and leg SAAT (40), or even, in accordance with Kuk et al. (37), from a phenotype associating an increase of abdominal SAAT, leg SAAT with a simultaneous reduction of VAT. Nevertheless, none of the studies carried out until now have proposed an anthropometric model which combines Waist C and either Thigh C or Hip C and which captures at best the heterogeneity of adipose tissues and their relationship to cardiovascular risk factors and diseases, especially after the emergence of interesting concepts such as the health protective effect of femoro-gluteal adiposity (39, 40).

In conclusion, our anthropometric models, simple and inexpensive, improve substantially VAT prediction in Europides (in the South of France) probably because they provide an independent estimation of VAT and SAAT, helping to differentiate abdominal fat compartments, in a large range of age and BMI. These models are extremely useful for research and clinical practice. Otherwise, in other populations, our models and/or concept should indeed be re-validated against the gold standard methods before use (MRI, CT-Scan) It will consequently be interesting to assess, as part of future studies, the ability of our models to predict metabolic and cardiovascular morbidity and mortality.

## Supplementary material

Supplementary material file (Word format) includes 1.Statistics relating to the VAT prediction models using 2 measures highly correlated with VAT and comparisons with our anthropometric models (Annexes A, B); 2.Models with SAD, the best VAT predictor, substituted for Waist C because of its easier feasibility and good prediction of VAT (Annexe C).
