# Supplementary material for: VAT=TAAT-SAAT: Innovative Anthropometric Model to Predict Visceral Adipose Tissue Without Resort to CT-Scan or DXA
Source: Obesity (Silver Spring). 2012 Sep 19;21(1):E41–50. doi: 10.1002/oby.20033 (PMC3618381; doi:10.1002/oby.20033)
Supplement: Supplementary file 1 [file oby0021-0E41-SD1.doc]

| Annex A |  | Model Parameters  (p < 10^-4^) | | | Variables Parameters | | *Colinearity Diagnostic* | | | |
| --- | --- | --- | --- | --- | --- | --- | --- | --- | --- | --- |
| Women |  | R | R^2^ | SEE | r _partial_ | p _partial_ | Condition Index | Variance Proportion | VIF | Tolerance |
| Model 1 | Waist C | 0.887 | 0.787 | 41.458 | 0.798 | < 10^-4^ | 1;7.26; 13.76 | 0;0.04;0.96 | 1.255 | 0.797 |
|  | Age |  |  |  | 0.542 | < 10^-4^ |  | 0.01;0.92;0.07 | 1.255 | 0.797 |
| Model 2 | Waist C | 0.890 | 0.791 | 41.300 | 0.315 | 0.008 | 1;7.78;  11.98;  43.59 | 0;0;0.01;0.99 | 10.053 | 0.099 |
|  | BMI |  |  |  | 0.149 | 0.221 |  | 0;0.01;0.05;0.93 | 8.818 | 0.113 |
|  | Age |  |  |  | 0.552 | < 10^-4^ |  | 0;0.78;0.01; 0.21 | 1.447 | 0.691 |
| Model 3 | Waist C | 0.898 | 0.807 | 39.736 | 0.451 | < 10^-4^ | 1;8.09;  9.55; 26.75 | 0;0.01;0;0.99 | 4.044 | 0.247 |
|  | SAD |  |  |  | 0.308 | < 10^-4^ |  | 0;0.05;0.21;0.74 | 3.923 | 0.255 |
|  | Age |  |  |  | 0.546 | < 10^-4^ |  | 0;0.97;0.02;0.01 | 1.262 | 0.793 |
| Model 4 | Waist C | 0.900 | 0.811 | 39.642 | 0.174 | 0.157 | 1;8.45;  10.65;  20.73;  51.27 | 0;0;0;0.01;0.98 | 12.356 | 0.081 |
|  | SAD |  |  |  | 0.304 | 0.012 |  | 0;0.03;0.17;0.69;0.11 | 3.934 | 0.254 |
|  | Age |  |  |  | 0.554 | < 10^-4^ |  | 0;0.75;0.05;0.04;0.16 | 1.458 | 0.686 |
|  | BMI |  |  |  | 0.140 | 0.255 |  | 0;0.01;0;0.21;0.78 | 8.842 | 0.113 |
| Model 5 | SAD | 0.897 | 0.804 | 39.982 | 0.600 | < 10^-4^ | 1;8.22;  9.07;  42.85 | 0;0.02;0.65;0.33 | 1.944 | 0.514 |
|  | WHR |  |  |  | 0.440 | < 10^-4^ |  | 0;0;0;0.99 | 2.585 | 0.387 |
|  | Age |  |  |  | 0.380 | 0.001 |  | 0;0.76;0.03:0.21 | 1.619 | 0.618 |
| Model 6 | SAD | 0.913 | 0.834 | 37.092 | 0.309 | 0.010 | 1;8.619;  9.817;  21.21;  48.36 | 0;0.04;0.18;0.68;0.1 | 3.498 | 0.286 |
|  | WHR |  |  |  | 0.388 | 0.001 |  | 0;0;0.01;0.01;0.99 | 2.728 | 0.367 |
|  | Age |  |  |  | 0.458 | < 10^-4^ |  | 0;0.66;0.05;0.06;0.23 | 1.673 | 0.598 |
|  | BMI |  |  |  | 0.390 | 0.001 |  | 0;0.04;0.01;0.93;0.02 | 3.054 | 0.327 |
| Model 7 | Waist C | 0.892 | 0.796 | 40.851 | 0.576 | < 10^-4^ | 1;8.107;  15.861;  50.929 | 0;0.01;0.43;0.57 | 3.045 | 0.328 |
|  | WHR |  |  |  | 0.208 | 0.086 |  | 0;0;0;1 | 3.929 | 0.255 |
|  | Age |  |  |  | 0.428 | < 10^-4^ |  | 0;0.73;0.05;0.21 | 1.642 | 0.609 |
| Model 8 | Waist C | 0.904 | 0.817 | 38.967 | -0.043 | 0.726 | 1;8.65;  12.57;  39.1;  84.92 | 0;0;0;0.11;0.89 | 22.638 | 0.044 |
|  | WHR |  |  |  | 0.351 | 0.003 |  | 0;0;0;0.15;0.85 | 5.622 | 0.178 |
|  | Age |  |  |  | 0.467 | < 10^-4^ |  | 0;0.71;0.01;0.26;0.02 | 1.656 | 0.604 |
|  | BMI |  |  |  | 0.322 | 0.007 |  | 0;0.01;0.05;0.27;0.68 | 12.618 | 0.079 |
| Model 9 | Waist C | 0.901 | 0.812 | 39.157 | 0.789 | < 10^-4^ | 1;7.26;  10.967;  13.896 | 0;0;0.35;0.42 | 2.352 | 0.632 |
|  | Proximal Thigh C |  |  |  | -0.348 | 0.003 |  | 0;0.02;0;0.45 | 1.909 | 0.724 |
|  | Age |  |  |  | 0.376 | 0.001 |  | 0;0.36;0.05;0.39 | 1.686 | 0.893 |
| Model 10 | Waist C | 0.914 | 0.836 | 36.881 | 0.313 | 0.009 | 1;7.95;  10.81;  12.5;  13.98 | 0;0;0.01;0.12;0.42 | 2.123 | 0.699 |
|  | Proximal Thigh C |  |  |  | -0.463 | < 10^-4^ |  | 0;0.01;0.01;0.28;0.19 | 2.352 | 0.725 |
|  | Age |  |  |  | 0.433 | < 10^-4^ |  | 0;0.27;0.01;0.39;0.03 | 1.715 | 0.683 |
|  | BMI |  |  |  | 0.355 | 0.003 |  | 0;0;0.06;0;0.24 | 1.862 | 0.792 |

**Annex A: In women, VAT prediction models using two measures both initially highly correlated with VAT in the univariate regression models. Comparisons with the (Waist C, Proximal Thigh C, age ± BMI) models (Models 9, 10)** (each model has a constant no described in this table)

| Annex B |  | Model Parameters  (p < 10^-4^) | | | Variables Parameters | | *Colinearity Diagnostic* | | | |
| --- | --- | --- | --- | --- | --- | --- | --- | --- | --- | --- |
| Men |  | R | R^2^ | SEE | r _partial_ | p _partial_ | Condition Index | Variance Proportion | VIF | Tolerance |
| Model 1 | Waist C | 0.874 | 0.764 | 51.631 | 0.833 | < 10^-4^ | 1;6.76;  14.83 | 0;0.07;0.92 | 1.04 | 0.961 |
|  | Age |  |  |  | 0.554 | < 10^-4^ |  | 0.01; 0.99; 0 | 1.04 | 0.961 |
| Model 2 | Waist C | 0.875 | 0.765 | 52.175 | 0.401 | < 10^-4^ | 1;6.83;  13.35;  46.51 | 0;0;0;0.99 | 9.494 | 0.105 |
|  | BMI |  |  |  | 0.066 | 0.681 |  | 0;0.01;0.05;0.94 | 9.129 | 0.110 |
|  | Age |  |  |  | 0.531 | 0.009 |  | 0;0.65;0.13;0.22 | 1.295 | 0.772 |
| Model 3 | Waist C | 0.876 | 0.767 | 51.986 | 0.835 | < 10^-4^ | 1;6.76;  9.5;  17.25 | 0;0;0.18;0.81 | 1.066 | 0.938 |
|  | SAD |  |  |  | -0.107 | 0.504 |  | 0;0.31;0.65;0.03 | 1.026 | 0.975 |
|  | Age |  |  |  | 0.556 | < 10^-4^ |  | 0.01;0.69;0.3;0.01 | 1.041 | 0.961 |
| Model 4 | Waist C | 0.876 | 0.768 | 52.520 | 0.402 | 0.010 | 1;7.37;  8.88;  15.73;  51.88 | 0;0;0.01;0;0.99 | 9.496 | 0.105 |
|  | SAD |  |  |  | -0.113 | 0.489 |  | 0;0.13;0.71;0.15;0 | 1.031 | 0.970 |
|  | Age |  |  |  | 0.536 | < 10^-4^ |  | 0;0.63;0.03;0.12;0.22 | 1.297 | 0.771 |
|  | BMI |  |  |  | 0.074 | 0.649 |  | 0;0;0.02;0.04;0.94 | 9.174 | 0.109 |
| Model 5 | SAD | 0.558 | 0.311 | 89.314 | -0.241 | 0.129 | 1;6.47;  11.35;  26.27 | 0;0.06;0.2;0.73 | 3.523 | 0.284 |
|  | TAD |  |  |  | 0.322 | 0.040 |  | 0;0.01;0;0.99 | 3.561 | 0.281 |
|  | Age |  |  |  | 0.458 | 0.003 |  | 0.01;0.68;0.31;0 | 1.023 | 0.977 |
| Model 6 | SAD | 0.856 | 0.749 | 54.670 | -0.312 | 0.050 | 1;7.19;  9.5;  15.66;  29.39 | 0;0.05;0.12;0.09;0.74 | 3.537 | 0.283 |
|  | TAD |  |  |  | 0.302 | 0.058 |  | 0;0;0.01;0.01;0.98 | 3.680 | 0.272 |
|  | Age |  |  |  | 0.655 | < 10^-4^ |  | 0;0.73;0.1;0.16;0.01 | 1.024 | 0.977 |
|  | BMI |  |  |  | 0.797 | < 10^-4^ |  | 0;0.01;0.44;0.54;0 | 1.064 | 0.940 |
| Model 7 | Waist C | 0.874 | 0.764 | 52.285 | 0.823 | < 10^-4^ | 1;7.32;  14.11;  17.96 | 0;0.02;0.55;0.42 | 1.132 | 0.883 |
|  | TAD |  |  |  | -0.012 | 0.942 |  | 0;0.04;0.72;0.23 | 1.101 | 0.908 |
|  | Age |  |  |  | 0.554 | < 10^-4^ |  | 0.01;0.97;0.02;0.01 | 1.043 | 0.958 |
| Model 8 | Waist C | 0.875 | 0.765 | 52.856 | 0.396 | 0.012 | 1;7.57;  11.96;  18.85;  52.49 | 0;0;0.01;0;0.99 | 9.840 | 0.102 |
|  | TAD |  |  |  | -0.005 | 0.974 |  | 0;0.01;0.36;0.61;0.02 | 1.111 | 0.900 |
|  | Age |  |  |  | 0.531 | < 10^-4^ |  | 0;0.68;0.07;0.04;0.21 | 1.295 | 0.772 |
|  | BMI |  |  |  | 0.065 | 0.689 |  | 0;0.01;0.04;0.01;0.94 | 9.217 | 0.108 |
| Model 9 | Waist C | 0.896 | 0.803 | 47.735 | 0.819 | < 10^-4^ | 1;6.79;  10.11;  11.87 | 0;0.01;0.33;0.41 | 2.381 | 0.620 |
|  | Proximal Thigh C |  |  |  | -0.408 | 0.008 |  | 0,0.01;0;0.08 | 2.566 | 0.790 |
|  | Age |  |  |  | 0.305 | 0.053 |  | 0;0.23;0;0.17 | 1.610 | 0.821 |
| Model 10 | Waist C | 0.904 | 0.817 | 46.687 | 0.452 | 0.003 | 1;7.18;  9.04;  10.74;  12;75 | 0;0;0.01;0.12;0.27 | 2.50 | 0.705 |
|  | Proximal Thigh C |  |  |  | -0.469 | 0.002 |  | 0;0.01;0.02;0.36;0.12 | 2.993 | 0.834 |
|  | Age |  |  |  | 0.351 | 0.026 |  | 0;0.28;0.03;0.15;0.04 | 1.667 | 0.600 |
|  | BMI |  |  |  | 0.261 | 0.104 |  | 0;0;0.06;0;0.33 | 2.650 | 0.794 |

**Annex B: In men, VAT prediction models using two measures both initially highly correlated with VAT in the univariate regression models. Comparisons with the (Waist C, Proximal Thigh C, age ± BMI) models (Models 9, 10)** (each model has a constant no described in this table)

| Annex C | VAT Prediction Model including SAD, Proximal Thigh C, Age and BMI | Model Parameters  (p < 10^-4^) | | |
| --- | --- | --- | --- | --- |
| In Women: | | R | R^2^ | SEE |
| VAT = 5,47*SAD - 3,52* Proximal Thigh C + 1,39* Age + 6,58*BMI - 22,48 | | 0.927 | 0. 859 | 34.25 |
|  | |  |  |  |
| In Men: | |  |  |  |
| VAT = 5,08*SAD - 4,24* Proximal Thigh C + 1,635* Age + 10,063*BMI - 66,123 | | 0.890 | 0.792 | 49.78 |

**Annex C: Visceral adipose tissue prediction by the multiple linear regressions associating SAD, Proximal Thigh C, Age and BMI.**
